# LGBTQIA+ STEM Day 2025: An interview with Fabrice Roux

**DOI:** 10.1038/s42003-025-09195-1

**Published:** 2025-11-18

## Abstract

To commemorate LGBTQIA+ STEM Day this year, *Communications Biology* is reaching out to discuss their personal and professional experiences in research. In this Q&A, we are talking to Dr. Fabrice Roux, a CNRS research director at Toulouse in France, who works on plant adaptation at the intersection of ecological genomics, quantitative genetics, and molecular biology.

**Figure:**
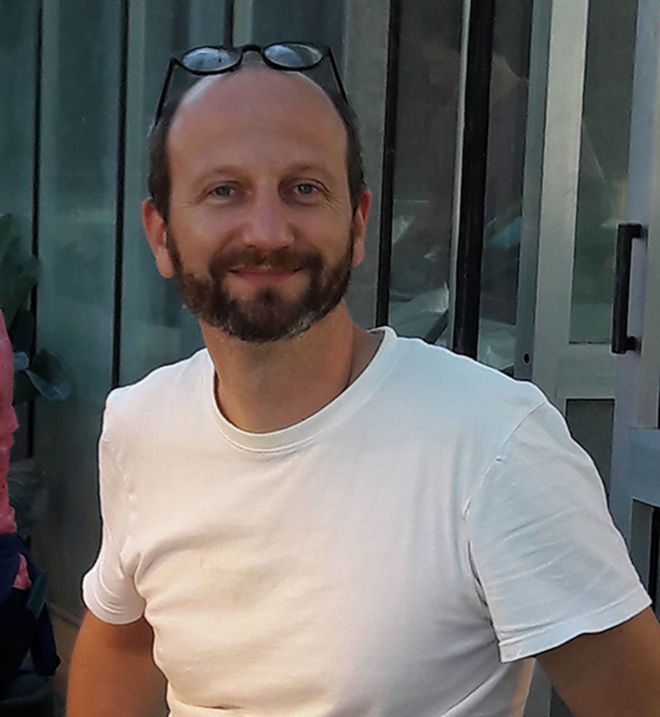
Claudette Icher

Just to get started, could you please tell me a little bit about your academic background?

Since I was in high school, I have always wanted to study how plants adapt to their environments. My parents were farmers, and I grew up in a very nice countryside in Burgundy, in France. I obtained a master’s degree in ecology and evolution at the University of Montpellier in 2000. I obtained a second master’s degree in plant biology at the University of Burgundy in 2001. During my master’s internships, I worked on the evolution and the genetic basis of natural variation of flowering time. Then, I started a PhD at the INRAE [Institut National de la Recherche Agronomique] institute in Dijon. During my PhD, I combined experiments and modeling to understand and predict the dynamics of herbicide resistance in crop fields. Then, in 2006, I spent one year at the University of Chicago in the group of Joy Bergelson, working on the evolutionary ecology and the genetic bases of plant-pathogen interactions. The year I was in Chicago, I applied for a CNRS position. I got it that first year, and so I had to come back to France in January 2007. Even if I only spent a single year at the University of Chicago, it was a fantastic time to be there, and I wish I could have spent more time, like 3 or 5 years. So, at the age of 29, I started my career as a CNRS researcher at the University of Lille in the Department of Ecology and Evolution. In 2012, the Department of Molecular Biology and Biochemistry, dedicated to the study of plant-microbe interactions in Toulouse, asked me to develop a team in ecology and evolution. I really liked this challenge, and in 2013, I moved to Toulouse. Since 2013, my team has been very successful in developing interdisciplinary projects between ecological genomics, quantitative genetics, evolutionary biology, and molecular biology, with a focus on understanding and predicting plant-microbiota-pathobiota interactions and plant-plant interactions in the context of global change. An example of this success is the obtention of a Synergy grant from the ERC (European Research Council), certainly one of the most prestigious grants in Europe, with my colleagues Detlef Weigel from the Max Planck Institute in Tübingen and Joy Bergelson from New York University.

You mentioned that you started off at the interface of plant ecology and evolution, and then a PhD on herbicide resistance. When you started your own research group, why did you decide to focus more on plant-plant or plant-microbe interactions?

This is a very interesting and important question. Since my first master’s internship, I have always had three major questions about adaptation in plants. What are the selective agents acting on plants? What is the genetic architecture of adaptation in plants? And what are the genetic and molecular mechanisms of adaptation in plants? Over the course of my career, I have shifted from studying adaptive traits for which the answers to these three questions were mostly available to studying adaptive traits for which few answers were available, mainly using *Arabidopsis thaliana* as a plant model. That’s why I started to work on herbicide resistance, because we know very well the selective agents and the genetic mechanisms underlying this adaptive trait. However, the number of studies reporting the dynamics of herbicide resistance in crop fields was very limited at the time of my PhD. Then, I moved to working on phenological traits, including flowering time. For this trait, the underlying genetic and molecular mechanisms are very well described. However, the identification of the selective agents acting on flowering time and the characterization of the genetic architecture of flowering time in complex environments required further studies conducted in the native environments of *A. thaliana*.

Finally, since I moved to Toulouse, I focused on adaptive traits, namely plant-microbiota-pathobiota interactions and plant-plant interactions, for which we have very few answers to the three questions mentioned above. Let’s take the example of plant-plant interactions. While *A. thaliana* has been described as a pioneer species and a poor competitor, we found that *A. thaliana* interacts with, on average, 12 other plant species in natural settings and can outcompete some species. We also recently cloned a gene likely involved in the active perception of neighboring species.

My future research will be to understand and predict how plants respond to multistress environments (climate, soil physico-chemical properties, microbiota, pathogens, competitors, urbanization…) in the context of global change, in particular by identifying the genetic and molecular mechanisms associated with multistress tolerance, with the lofty goal of translating my research to crops.

How has your toolkit for this research changed over time? Are there any techniques that you’re particularly excited about these days?

I’m really excited by the recent development in long-read sequencing technologies. For instance, in my team, we recently established a collection of more than 1300 bacterial strains isolated from the phyllosphere of *A. thaliana* collected in 168 natural populations located in the southwest of France. Using the Nanopore technology, we have sequenced the genomes of 400 bacterial strains in less than a week. I also collected thousands of accessions of *A. thaliana*, mainly in France. We recently started to sequence them with long-read sequencing technologies.

Having access to the full genomes of bacterial strains and *A. thaliana* accessions represents a fantastic opportunity to explore the pangenome at the within-species level and the meta-pangenome at the interspecific level. In particular, we can estimate the relative importance of structural variants in the adaptation of *A. thaliana* and its bacterial species composing its microbiota to complex abiotic and biotic environments. For instance, based on a new statistical approach that I developed, I identified several bacterial genes associated with climate variation. To my knowledge, no studies have reported the genetic basis of adaptation to climate in bacteria despite their presence in almost every habitat on Earth. Even more intriguingly, we found evidence that some candidate genes for climate adaptation may have been transferred between bacterial phyla.

The last development in my team is based on combining artificial intelligence with pangenome or meta-pangenome data. The goal is to develop bacterial synthetic communities and plant synthetic communities, which are highly performant while being robust to environmental perturbations.

It seems like you have some very interdisciplinary projects going on.

Yes, definitely. This is what I like.

As an aside, the term “meta-pangenome” is new to me. I guess it would be redundant to call it the “pan-pangenome.”

Yes.

Changing gears, one reason I reached out is because I saw your profile on 500 Queer Scientists. How do you think being a queer scientist has factored into your career?

It has been very difficult for me, and I want to be honest about that. When studying and working in France, I got a lot of questions about my sexual orientation. I don’t think it was appropriate to ask me these kinds of questions. Even worse, I received several homophobic comments from different colleagues. I recently decided to move to another country to feel safe when I go to work. Obviously, it has been a very hard decision to quit the team that I created in 2013, and it will be a lot of work to transfer all my research projects to a new place. But I need to first think about my health.

I’m really sorry to hear that you’ve been put through that situation. As a gay man myself, I think there is a weird balance in terms of how much of your identity you’re expected to disclose to others. When I was looking for a lab as part of my PhD, or jobs after that point, I was never asked about my sexuality. But at the same time I felt obligated to bring it up, because I was worried about whether it would be some kind of “problem” later on.

Exactly. At the CNRS, I’m unfortunately not the only one to have been the target of LGBTphobia. For instance, LGBTQIA+ members of another lab in Toulouse complained about the absence of help from the HR department. In addition, every year, there is a training in LGBT leadership in Berlin. I asked twice to the CNRS if I could follow the training, and was told no by the HR department.

I was invited to participate in a webinar dedicated to the quality of life of PhD students. During this webinar, I talked about being a victim of homophobic comments. On the chat, two PhD students wrote to me and told me they had similar experiences, but they didn’t know what to do, etc. There had been a recent survey in France showing that 20% of French people between the ages of 15 and 25 say they belong to the LGBTQIA+ community. So 20% is not like a minority anymore.

Based on my experience and the experience of other colleagues of the LGBTQIA+ community, including PhD students, I realized that I have to do something to try to stop discrimination against the LGBTQIA+ community in French universities and research institutes. So, in 2022, I contacted all the other French people who were on the 500 Queer Scientists website. Sadly, there were only four. After a few meetings, we said, “OK, maybe it would be good to write a charter against discrimination toward the LGBTQIA+ community”. So, we wrote a charter! We asked for advice from people working in social and human sciences who were actually working on this topic, and we wrote a charter to act against discrimination toward the LGBTQIA+ community in French academia. Nearly 600 people have signed the charter. This was overall highly positive, even if I received some very rude comments from colleagues from France, who thought that this charter was inappropriate. Following the publication of this charter, I was invited by the Pasteur Institute to talk about it. It was really nice to see that some people, some institutes, are eager to work on a better inclusion of the LGBTQIA+ members in French universities and research institutes. Unfortunately, my own employer, the CNRS, never mentioned this charter and has never asked me to talk about it.

The last thing I can mention is related to my participation in an evaluation panel for the ERC two years ago. Before we started evaluating projects in Brussels, the head of the ERC (Maria Leptin) talked for 30 minutes about being aware of bias that can occur unconsciously during our evaluations. In particular, she talked about bias against women, and then asked if we had any questions. I raised my hand and asked whether she was aware of any bias against the LGBTQIA+ community. She replied that the ERC doesn’t have any data about that, so they don’t know if there is a bias. Then, a colleague in my panel, who is also part of the LGBTQIA+ community, told her that it’s not because there is no data that we cannot do anything. A colleague from the social and human science panel indicated that some recent articles showed that researchers from the LGBTQIA+ community tend to be more creative, more inclusive, with a different way of thinking, which altogether makes it better for science to have LGBTQIA+ people in universities or research institutes. Maria Leptin was really open about the discussion and said a meeting should be set up to talk about this topic with the Gender & Diversity committee of the ERC. We had this meeting when we were in Brussels. The Gender & Diversity committee asked me if members of the LGBTQIA+ community would agree to tick a box indicating their membership in this community. I replied that we talked about this opportunity during a meeting with French colleagues, and this is clearly what we were asking for!

Have these experiences impacted how you manage your own research group?

I use a horizontal management approach. Everyone is free to speak. I know that some people want to do a PhD or postdoc in my group because I’m totally open, as a member of the LGBTQIA+ community. They feel safe about that, and they know I would protect them.

We talked about 500 Queer Scientists, but are there any other LGBTQIA+ initiatives you’ve been involved in, or would like to highlight?

No, I’m not aware of anything else. But I am more than open to participating in other LGBTQIA+ initiatives.

For a few years now, our journal has done features on members of the LGBTQIA+ community. I think it’s really important to highlight different experiences so that people can feel like they can be themselves in science and academia as a whole. So thank you for sharing your story as part of this series.

When I was participating in an evaluation panel for the ERC two years ago, I remember that a member of the panel told me that it was really great that I was inspired to talk about the LGBTQIA+ community. In fact, her son was also from the LGBTQIA+ community, and it was hard for him to join in science. In line, I also know that there is a report in France showing that LGBT phobia is more important in research institutes and universities than in regular life. I try to talk about my sexual orientation and my husband naturally. If people don’t like it, I don’t care anymore. I just want to make our workspace safer for the LGBTQIA+ community and I expect to be a role model for the next generation.

Before we wrap up, is there a question that you wish that I asked you during this this Q&A. Or do you have any other closing remarks?

I believe there is hope for greater inclusion of the LGBTQIA+ community in society, and I will always stand up for those within this community who work in universities and research institutes. I have no reason to be ashamed of who I am. We may not all agree on everything, but mutual respect is essential. This is what truly makes a society better.

*This interview was conducted by Deputy Editor, George Inglis*.

